# Robotic-assisted thoracoscopic resection of anterior mediastinal cystic teratoma: a case report and literature review

**DOI:** 10.1186/s13019-022-01806-w

**Published:** 2022-04-05

**Authors:** Harry Ramcharran, Jason Wallen

**Affiliations:** Department of Thoracic Surgery, SUNY Upstate Medial University, 750 East Adams Street, Syracuse, NY 13210 USA

**Keywords:** Teratoma, Mediastinum, Single lung ventilation, Double lung ventilation

## Abstract

**Background:**

Mediastinal teratomas are rare tumors that frequently occur in the anterior mediastinum. The majority of these tumors are benign and slow growing. Due to their low malignant potential, the treatment for these tumors is surgical resection. More recently, the surgical management has shifted from invasive approaches such as a sternotomy to minimally invasive ones such as robotic-assisted thoracoscopic resections utilizing lung isolation ventilation. We present a rare case of a locally advanced mediastinal teratoma requiring resection, which was initially attempted thoracoscopically using double lung ventilation.

**Case presentation:**

A 43 year-old female was found to have an anterior mediastinal mass during work-up for an intermittent cough in 2009. Chest imaging and biopsy at the time showed evidence of a cystic teratoma without concerning features. She underwent imaging surveillance until 2018, when repeat chest imaging showed increasing growth and worrisome radiologic features concerning for malignant degeneration. She underwent an elective robotic-assisted thoracoscopic resection utilizing double lung ventilation, but due to extensive involvement of the right lung, pericardium, superior vena cava, and right phrenic nerve the patient had to be repositioned and started on single lung ventilation mid-procedure to facilitate a safe and complete resection.

**Conclusions:**

Anterior mediastinal teratomas can be successfully removed by robotic-assisted thoracoscopic resections utilizing single lung ventilation. Though robotic-assisted thoracoscopic resection utilizing double lung ventilation can be effective in performing certain procedures such as lung wedge resections, thymectomy, pleural biopsies and minimally invasive cardiac procedures, it is limited in removing locally advanced mediastinal tumors.

## Background

Extra-gonadal teratomas of the mediastinum are very rare tumors that occur most frequently in the anterior mediastinum. The majority of these tumors are benign and slow growing, and as a result, are found incidentally [1–3]. Due to their low malignant potential, the treatment for these tumors is surgical resection, which affords excellent long-term disease-free survival [3]. Though many cases of mediastinal teratomas have been reported, we present a rare case of a locally advanced mediastinal teratoma requiring an extensive robotic-assisted thoracoscopic resection of the pericardium, lung, superior vena cava, and phrenic nerve. Many of these robotic-assisted resections are performed with the patient in supine position and utilizing single lung ventilation. Given the success of double lung ventilation during thoracoscopic lung wedge resection and thymectomy, we attempted the resection with the patient positioned supine and with double lung ventilation in order to avoid the complications of using a dual-lumen endotracheal (ET) tube as well as decreasing the overall operative time.

## Case presentation

**Fig. 1:**
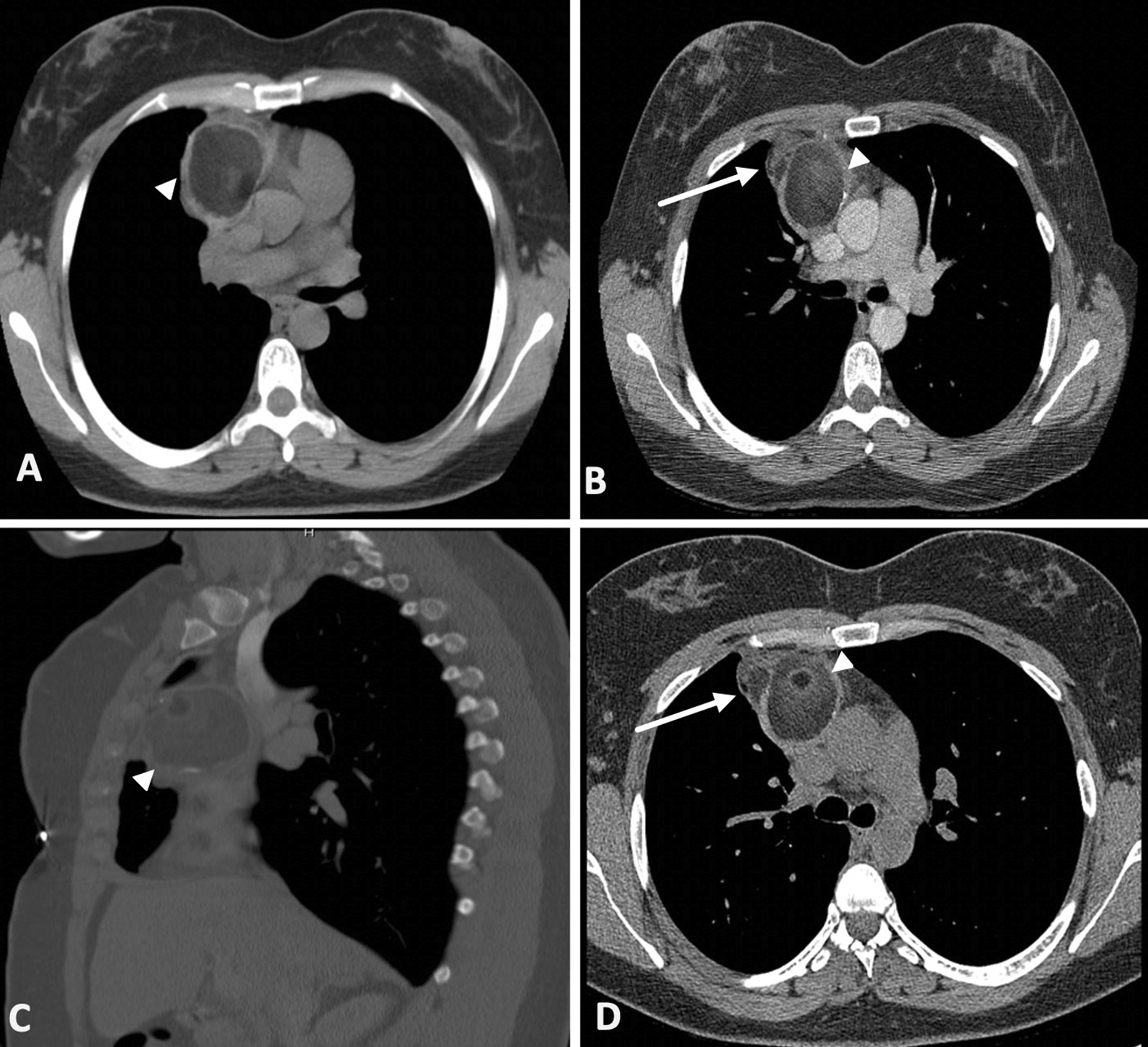
Initial axial computerized tomography (CT) of the thorax in 2014 showing mediastinal mass (**A**). Axial (**B**) and sagittal (**C**) CT of the thorax in 2018 showing increase in size of heterogenous mass with a new lobulation (arrow). Axial CT thorax prior to resection (**D**) showing an increase in size of the new lobulation (arrow)

A 43 year-old-female was found to have an anterior mediastinal mass while being worked up for an intermittent cough in 2009. Further workup with computerized tomography (CT) of the thorax and CT guided percutaneous biopsy showed a benign cystic teratoma with negative tumor markers. The patient subsequently underwent yearly surveillance CT scans. A surveillance CT Thorax done in 2014, showed the anterior mediastinal mass to measure 5.2 × 5.0 × 5.5 cm and composing mostly of fat with some mural calcification and a thickened capsule (Fig. 1A). Subsequent surveillance scans showed a stable mass for which, she presented to our clinic in 2018 for cessation of surveillance. However, repeat CT thorax showed the mass to have enlarged (5.4 × 4.8 × 6.2 cm) with a new lobulation extending to the right of the initial lesion, measuring 15 mm (Fig. 1B, C). Due to the further increase in size of the mass and new lobulation (15–19 mm) (Fig. 1D) there was a concern for malignant degeneration, as such, the plan was for surgical excision of the mass.

**Fig. 2:**
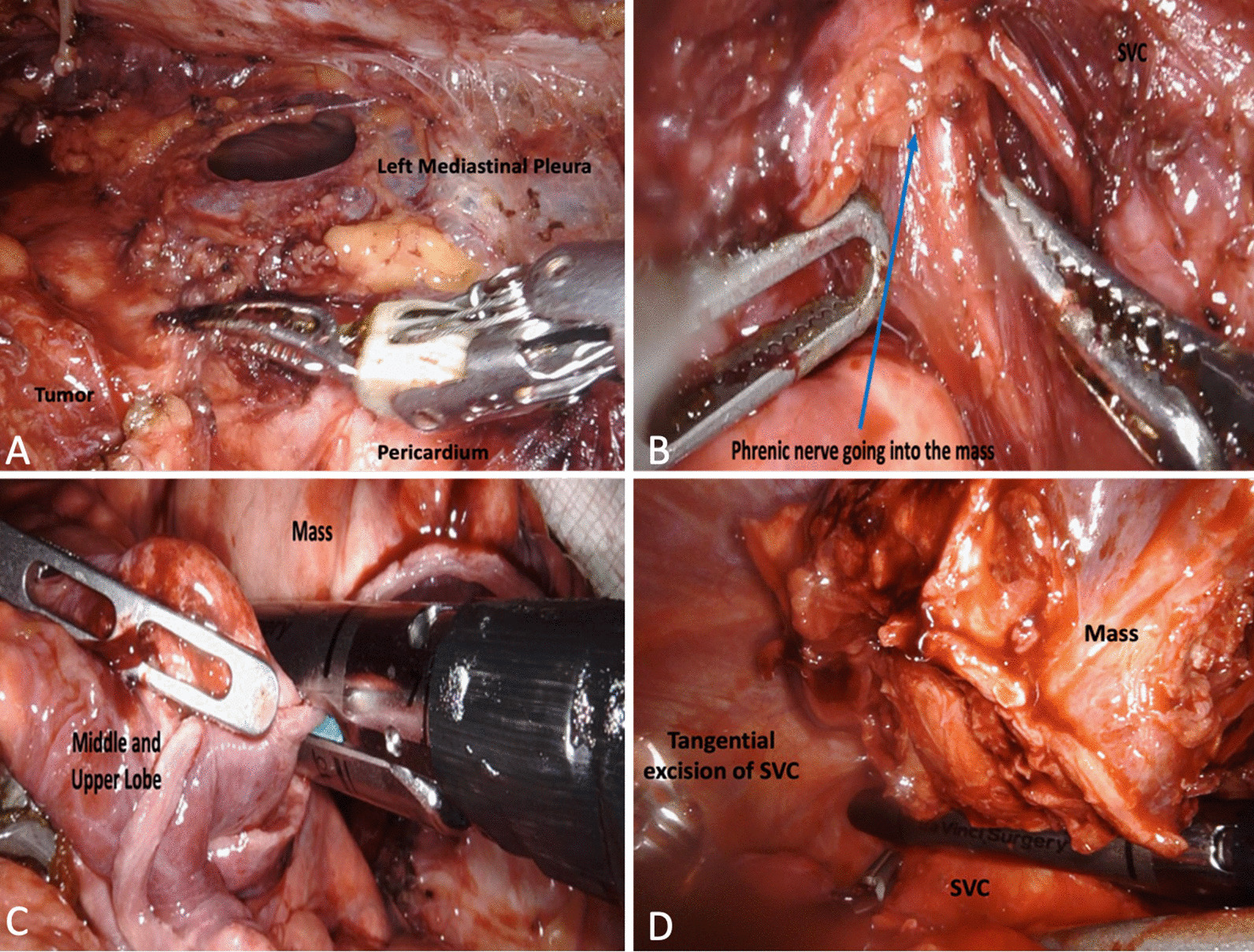
Intraoperative images during initial lateral approach depicting dissection of mass from left mediastinal pleura and pericardium (**A**), phrenic nerve entering body of the tumor (**B**). Intraoperative images with patient in left lateral decubitus position depicting wedge resection of right middle and upper lobes (**C**) and tangential excision of the SVC (**D**)

The planned procedure was a robotic-assisted thoracoscopic resection via right lateral approach. The patient was brough to the operating room, placed supine, and general anesthesia was started. A single lumen ET tube was initially used to intubate the patient in order to decrease operative time, and minimize the complications associated with a dual lumen ET tube and general anesthesia. In addition, on pre-operative CT thorax, there were no overt signs of lung and mediastinal structure invasion that would have precluded the use of double lung ventilation. The thorax was then entered via standard thoracoscopic incisions and a total of 3 8 mm ports were used (6th intercostal space in the anterior axillary and mid-clavicular line, and the 4th intercostal space in the anterior axillary line). Upon initial inspection, the mass was adherent to the right upper lobe and the right phrenic nerve. Dissection began medially with a combination of blunt dissection and electrocautery. The mass was dissected from the anterior chest wall but found to be abutting the innominate vein and densely adhered to the superior vena cava (SVC). The mass was meticulously dissected free from the anterior pericardium, innominate vein, and aortic arch (Fig. 2A). The right phrenic nerve was found to be circumferentially encapsulated by the mass and was sacrificed (Fig. 2B). Anterior dissection continued until it was deemed difficult to assess the full extent of anterior lung and SVC involvement. The decision was then made to change the patient position to left lateral decubitus and utilize single lung ventilation.

The robotic instruments were removed from the patient and the robot was undocked. The single lumen ET tube was exchanged for a double lumen one to facilitate single lung ventilation. The patient was then placed in the left lateral decubitus position. Four additional 8 mm ports were placed along the 7th intercostal space and dissection continued posteriorly and laterally. The mass was attached to portions of the right upper and middle lobe, for which 2 wedge resections were done to free the mass from the respective lung lobes (Fig. 2C). The tumor was found to be too adhered to the SVC, as such, a tangential resection of the mass with the SVC was done with a vascular load using the robotic stapler (Fig. 2D). After the mass was freed circumferentially, it was removed by a specimen bag. After specimen retrieval, a right robotic diaphragmatic plication was done and a 28 French chest tube was left in place.

On gross examination, the tissue specimen was tan-pink in color with a cystic cavity filled with tan-gray gummous material as well as a fatty nodule. Histopathological examination revealed a mature cystic teratoma with an extensive cystic component with negative margins. The patient had an uneventful post-operative course and was discharged on post-operative day 3.

## Discussion

Teratomas are the most common germ cell tumor of the anterior mediastinum. These tumors are often very slow growing and are typically found incidentally on chest x-rays during unassociated work-up [1–5]. Teratomas often consist of tissue derived from all three embryonal germ cell layers and can be classified as mature, well differentiated, poorly differentiated, or immature with malignant potential [6, 7]. On CT imaging, they are a heterogenous appearing mass with fluid, fat, and calcification [3]. When symptoms do arise, though there are uncommon, they are often due to compression of adjacent mediastinal structures. Patients will typically present with symptoms of cough, dyspnea, chest pain, and post-bronchial pneumonia [5, 8]. The treatment of choice is surgical resection, which has shown excellent long-term disease-free survival [2–5].

The traditional surgical approach for resection of an anterior mediastinal germ cell tumor has been via a median sternotomy [8, 9]. However, the morbidity and mortality associated with a sternotomy has pushed surgeons towards video-assisted thoracoscopic (VATS) or robotic-assisted thoracoscopic surgery [2, 10]. During these procedures patients are often placed in the lateral decubitus or supine positions, with the port placements being more anterior on the chest wall with supine positioning. Though thoracoscopic lung resections are commonly performed with single lung ventilation (SLV), if there is no anticipated lung involvement of the mediastinal mass, then double lung ventilation (DLV) can provide adequate exposure and decrease undesired complications of using a dual lumen ET tube [11]. DLV with carbon dioxide insufflation has been used for procedures such as thoracoscopic pleural biopsies, lung wedge resections, thymectomy, and minimally invasive repair of atrial septal defects [11, 12]. A single center retrospective study comparing SLV and DLV for primarily pulmonary wedge and mediastinal mass resections have shown decreased operative time, and time to incision in the DLV group. Moreover, there were no differences in complications such as hemodynamic compromise between the groups [13]. Though the feasibility and safety of DLV for certain thoracic procedures has been established [12], the hesitancy in its application for more thoracoscopic procedures is due to concerns of a potential tension pneumothorax developing from carbon dioxide insufflation. However, patients are often able to tolerate up to 10-15 mmHg of intrathoracic pressure without any hemodynamic compromise [13]. Similarly, in a retrospective study of patients undergoing minimally invasive atrial septal defects with either SLV or DLV, Sen et al. [14] showed that DLV resulted in decrease operative time, intensive care and hospital stay, and complications such as re-expansion pulmonary edema. Moreover, they showed a higher first-pass intubation rate with DLV (89.9%) compared to SLV (73.9%), with two patients in the SLV group developing significant airway edema requiring ICU level of care [14]. These two studies support the trend towards minimally invasive thoracoscopic resections along with the selective use of double lung ventilation in order to reduce operative time, cost, and complications associated with a dual lumen ET tube.

This case highlights the successes of performing a minimally invasive thoracoscopic resection of an anterior mediastinal tumor as well as the limitations of using double lung ventilation. Performing this resection via a median sternotomy provides the best anatomic exposure as teratomas can be large and invasive [14, 15] however, thoracoscopic resection minimizes the morbidity associated with a median sternotomy with equivalent outcomes [16]. In a retrospective review comparing VATS with double lung ventilation vs sternotomy for early stage thymoma, Pennathur et al. [17] showed comparable oncological outcomes and decreased hospital stay in the VATS group. Similarly, Toolabi et al. [11] showed that the use of DLV in patients undergoing minimally invasive thoracoscopic thymectomy or sympathectomy did not result in periods of desaturation, hemodynamic instability, or peri-operative complications. Furthermore, they concluded that using SLV is not required during thoracoscopic surgery when lung resection is not anticipated or in select patient population with poor pulmonary reserve and multiple comorbidities [11]. In this case, we attempted resection with the patient supine and with DLV in order to facilitate a safe dissection with minimal OR time and without the associated complexity of a dual lumen ET tube. Placement of a dual lumen ET tube can require multiple intubation attempts resulting in complications such as tracheobronchial tree injury, hypoxemia, bleeding, and bronchospasms [14]. However, intraoperatively, the extent of the anterior mediastinal involvement of the teratoma was more than that seen on pre-operative CT scans, as the tumor was found to be invading the anterior upper and middle lung lobes, right phrenic nerve, and the SVC. As such, we decided to undock the robot, reposition the patient, and use single lung ventilation with a dual lumen ET tube in order to better assess the anterior lung involvement and facilitate a safe and complete resection. The risk of an incomplete resection or inadvertent damage to mediastinal structures was greater than the complications associated with SLV.

## Conclusions

There is no consensus on the optimal approach to resection of mediastinal teratomas but a robotic-assisted thoracoscopic resection is a feasible approach to a benign teratoma with regards to reduced operative time and improved peri-operative outcomes. Though DLV has been used for lung wedge resections, thymectomy, and pleural biopsies, using DLV during resection of a densely adherent anterior mediastinal mass can make assessing the extent of involvement and a safe resection challenging.

## Data Availability

The data used is available upon request from the corresponding author.

## Abbreviations

CT: Computerized tomography
SLV: Single lung ventilation
DLV: Double lung ventilation
SVC: Superior vena cava
